# Lessons learned 1 year after SARS-CoV-2 emergence leading to COVID-19 pandemic

**DOI:** 10.1080/22221751.2021.1898291

**Published:** 2021-03-22

**Authors:** Kelvin Kai-Wang To, Siddharth Sridhar, Kelvin Hei-Yeung Chiu, Derek Ling-Lung Hung, Xin Li, Ivan Fan-Ngai Hung, Anthony Raymond Tam, Tom Wai-Hin Chung, Jasper Fuk-Woo Chan, Anna Jian-Xia Zhang, Vincent Chi-Chung Cheng, Kwok-Yung Yuen

**Affiliations:** a State Key Laboratory of Emerging Infectious Diseases, The University of Hong Kong, Pokfulam, Hong Kong Special Administrative Region, People’s Republic of China; b Department of Microbiology, Li Ka Shing Faculty of Medicine, The University of Hong Kong, Pokfulam, Hong Kong Special Administrative Region, People’s Republic of China; c Carol Yu Centre for Infection, Li Ka Shing Faculty of Medicine, The University of Hong Kong, Pokfulam, Hong Kong Special Administrative Region, People’s Republic of China; d Department of Microbiology, Queen Mary Hospital, Pokfulam, Hong Kong Special Administrative Region, People’s Republic of China; e Department of Medicine, Li Ka Shing Faculty of Medicine, The University of Hong Kong, Pokfulam, Hong Kong Special Administrative Region, People’s Republic of China

**Keywords:** Coronavirus, COVID-19, SARS-CoV-2, Pandemic, Pathogenesis, Diagnostics, Treatment, Vaccines

## Abstract

Without modern medical management and vaccines, the severity of the Coronavirus Disease 2019 (COVID-19) pandemic caused by severe acute respiratory syndrome (SARS) coronavirus 2 (SARS-CoV-2) might approach the magnitude of 1894-plague (12 million deaths) and 1918-A(H1N1) influenza (50 million deaths) pandemics. The COVID-19 pandemic was heralded by the 2003 SARS epidemic which led to the discovery of human and civet SARS-CoV-1, bat SARS-related-CoVs, Middle East respiratory syndrome (MERS)-related bat CoV HKU4 and HKU5, and other novel animal coronaviruses. The suspected animal-to-human jumping of 4 betacoronaviruses including the human coronaviruses OC43(1890), SARS-CoV-1(2003), MERS-CoV(2012), and SARS-CoV-2(2019) indicates their significant pandemic potential. The presence of a large reservoir of coronaviruses in bats and other wild mammals, culture of mixing and selling them in urban markets with suboptimal hygiene, habit of eating exotic mammals in highly populated areas, and the rapid and frequent air travels from these areas are perfect ingredients for brewing rapidly exploding epidemics. The possibility of emergence of a hypothetical SARS-CoV-3 or other novel viruses from animals or laboratories, and therefore needs for global preparedness should not be ignored. We reviewed representative publications on the epidemiology, virology, clinical manifestations, pathology, laboratory diagnostics, treatment, vaccination, and infection control of COVID-19 as of 20 January 2021, which is 1 year after person-to-person transmission of SARS-CoV-2 was announced. The difficulties of mass testing, labour-intensive contact tracing, importance of compliance to universal masking, low efficacy of antiviral treatment for severe disease, possibilities of vaccine or antiviral-resistant virus variants and SARS-CoV-2 becoming another common cold coronavirus are discussed.

## The chronology of the pandemic

An outbreak of acute community-acquired atypical pneumonia of unknown aetiology was reported in Wuhan, the capital of Hubei province in central China, in December 2019. The initial cluster of cases was related to the Huanan seafood wholesale market where wild game animals were also sold [1]. During subsequent investigation, severe acute respiratory syndrome (SARS) coronavirus 2 (SARS-CoV-2) was detected in 33 out of 585 environmental samples taken from the market [2]. However, 45% of the cases with onset before 1 January 2020 had no apparent link to this market [3]. Retrospective molecular clock inference studies using phylogenetic analysis suggested that the earliest cases likely emerged between October and November 2019 [4, 5]. The culprit virus was identified using next-generation sequencing (NGS) on bronchoalveolar lavage fluids of three Wuhan patients [6]. The complete genome sequences of SARS-CoV-2 clustered in a distinct clade from SARS-CoV within the genus *Sarbecovirus*. The draft genome sequence was released on 10 January 2020, 10 days after the outbreak was announced.

As an escalating number of local cases was reported in Wuhan, a family cluster was identified in Shenzhen, a city in southern China 550 miles from Wuhan, between 10 and 15 January 2020 [7]. Six members of this family had returned from a trip to Wuhan between 29 December 2019 and 4 January 2020. Two of them visited a local hospital where a paediatric relative was hospitalized for pneumonia. Five of these six family members were clinically and/or virologically diagnosed with Coronavirus Disease 2019 (COVID-19) after returning to Shenzhen. In addition, a seventh family member, who did not go to Wuhan or visit wet markets in the preceding 14 days, became infected after staying in the same household with infected relatives. This familial cluster provided clear evidence of person-to-person transmission and inter-city spread by air travel. Furthermore, the report of an imported case in Thailand on 13 January 2020, and subsequently other countries suggested that global dissemination might have occurred earlier by frequent air travel.

**Figure 1.**
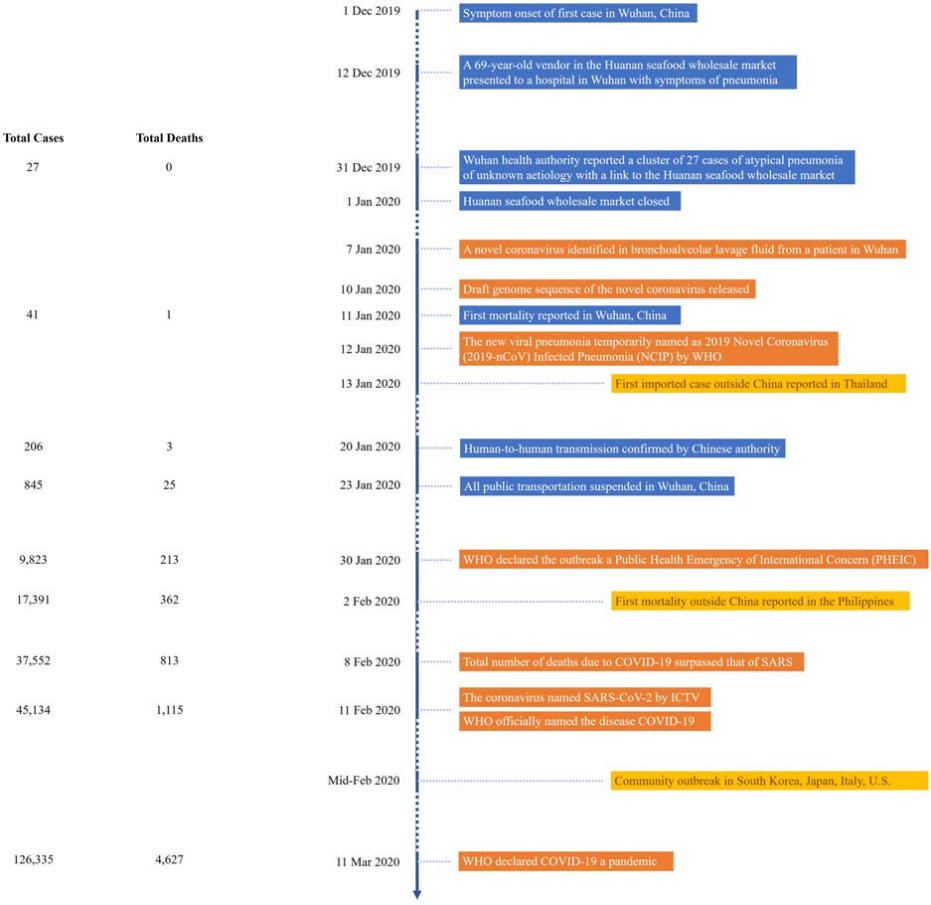
Chronology of events leading to the COVID-19 pandemic.

By the end of January 2020, SARS-CoV-2 was reported in 31 provinces in China, across East and Southeast Asia, and to Europe and the United States. Community transmission was detected in other Asian countries, a large part of Europe, the Middle East and the United States since February 2020 [8–11]. Figure 1 gives a detailed account of the unfolding of the pandemic. By April 2020, the total number of COVID-19 cases surpassed 1 million as more and more countries entered partial or nation-wide lock down. The death toll due to COVID-19 reached 1 million on 25 September 2020. By 22 December 2020, with the Chilean army reporting 36 cases at its research station in Antarctica, COVID-19 cases have been reported to affect all seven continents [12]. As of 4 February 2021, there have been more than 103 million confirmed cases with over 2 million deaths [13].

## Taxonomy, genomic organization, and replication cycle of SARS-CoV-2

SARS-CoV-2 belongs to the *Betacoronavirus* genus of the family *Coronaviridae*. This genus also includes the human respiratory pathogens SARS-CoV-1, Middle East respiratory syndrome (MERS) coronavirus (MERS-CoV), human coronavirus (HCoV)-HKU1, and HCoV-OC43 [14]. Together with the closely related bat coronavirus RaTG13 and SARS-CoV-1, SARS-CoV-2 is classified as a member of the *Sarbecovirus* subgenus of SARS-related coronaviruses [15]. Rapid characterization of SARS-CoV-2 by electron microscopy and NGS confirmed that it shared the structural features and genomic organization of other betacoronaviruses [6, 7]. It is a pleomorphic enveloped virus (size range: 60–140 nm) studded with distinctive surface spikes. The positive-sense single-stranded RNA genome of SARS-CoV-2 is around 29–30 kB in size and is organized as methyl-capped-5’UTR-ORF1a/b-S-ORF3-E-M-ORF6-ORF7a/b-ORF8-N/ORF9b-ORF10-3’UTR-poly A tail [16] (Table 1). One of the earliest published genome, hCoV-19/Wuhan/IVDC-HB-01/2019 (GISAID accession number (EPI_ISL_402119), has a genome size of 29,891 bp. A study using ribosome profiling techniques showed the presence of additional upstream and internal open reading frames (ORFs) [17]. The genome lacks the hemagglutinin esterase gene found in some other betacoronaviruses. ORF1ab, which comprises two-thirds of the entire genome, encodes a large polyprotein pp1ab, which is proteolytically cleaved into 16 non-structural proteins (Nsps) critical for viral replication [16]. Towards the 3′ end of the genome, the S, E, M, and N genes encode key structural proteins found in the mature virion [18]. The spike (S) glycoprotein forms trimers on the virion surface and binds to human angiotensin-converting enzyme 2 (ACE2) receptor for cell entry [19]. It contains two subunits S1 and S2 with a polybasic site PRRA at the junction, which enables effective cleavage by furin and other proteases [5]. This multibasic cleavage site appears to be an important virulence factor which may enhance virus replication and multiple tissue tropism as in the case of avian influenza A(H5N1) virus [20, 21]. Mutations in this site can attenuate pathogenicity in animal models and may be an attractive option for designing live attenuated vaccines [21]. Another cleavage site, called S2’, is located within the S2 region, and is cleaved by the transmembrane serine protease 2 (TMPRSS2) [22]. S protein contains major immunogenic epitopes, particularly concentrated in the N-terminal domain (NTD) and receptor binding domain (RBD) of the S1 subunit, which are targets of neutralizing antibodies. The envelope (E) protein likely forms a viroporin, which is important for virus assembly and release, and is also a putative virulence determinant [23]. The membrane (M) protein is an abundantly expressed structural protein within the lipid envelope that is also important for viral morphogenesis and interferon suppression [24]. Finally, the nucleocapsid protein (N) stabilizes the RNA genome in a helical complex [25] and serves as a key target of adaptive immunity. In addition, there are a number of accessory proteins, the function of some of which remains unknown. ORF3a may function as an inducer of apoptosis [26]. Both ORF6 and ORF8 are involved in interferon antagonism while ORF7a may be involved in inhibiting cellular translation [27–29]. ORF8 can bind to IL-17 receptor A (IL17RA) which may modulate the inflammatory response, and higher blood levels of soluble IL17A has been associated with milder disease [30]. Interestingly, circulating variants with loss-of-function deletions in SARS-CoV-2 ORF3b, ORF7a/7b, and ORF8 have been found, indicating that these are not absolutely essential for viral infection and may be remnants required for infection of an unidentified intermediate host [31–34]. ORF9b, an accessory protein translated from an alternative open reading frame within the N gene, interacts with the host mitochondrial import receptor protein TOM70 and suppresses type I interferon response [30, 35]. As for ORF10, it appears dispensable for cellular infection [36, 37].

**Table 1.** SARS-CoV-2 gene products.

Gene product	Putative primary function	Role in pathogenesis
Nsp1	Inhibit host protein translation; Degradation of host mRNA and disruption of mRNA export machinery to inhibit host gene gene expression	Suppression of interferon response
Nsp2	Unknown	
Nsp3	Polyprotein processing, de-ADP ribosylation, deubiquitination, interferon antagonist, formation of double membrane vesicles	
Nsp4	Formation of double membrane vesicles associated with replication complexes	
Nsp5	3C-like protease domain, polyprotein processing	Inhibit interferon signalling
Nsp6	Formation of double membrane vesicles associated with replication complexes	Interferon antagonist
Nsp7	Accessory subunit of RNA-dependent RNA polymerase	
Nsp8	Accessory subunit of RNA-dependent RNA polymerase; primase or 3′ terminal adenylyltransferase	
Nsp9	RNA-binding protein with a peptide binding site [48]	
Nsp10	Co-factor of nsp14 and nsp16 for methyltransferase activity	Interacts with NF-κB-repressing factor to facilitate interleukin-8 (IL-8) induction, which potentially increase IL-8-mediated chemotaxis of neutrophils and overexuberant host inflammation [49]
Nsp11	Unknown	
Nsp12	RNA-dependent RNA polymerase, nucleotidyltransferase	
Nsp13	Helicase RNA 5′ triphosphatase	Potent interferon antagonist
Nsp14	Proof-reading exonuclease RNA cap formation guanosine N7-methyltransferase	Potent interferon antagonist
Nsp15	Endoribonuclease Interferon antagonist	Potent interferon antagonist
Nsp16	Ribose 2′-O-Methyltransferase, RNA cap formation	
S	Binds to host cell receptor	
ORF3a		Induce apoptosis [26]
ORF3b		Interferon antagonist [33]
E	Envelope forms a homopentameric cation channel	May conduct Ca2+ out of the ERGIC lumen to activate the host inflammasome [23]
M	Membrane	Inhibit type 1 and III interferon production by direct interaction with RIG-I/MDA-5 and impeding downstream signalling [24]
ORF6		Potent interferon antagonist (block STAT1 and STAT2 nuclear translocation) [29]
ORF7a/b	Unknown	
ORF8	Downregulation of MHC-1, binds IL-17RA	Inhibit interferon pathway
N	Viral RNA genome protection and packaging, Virus particle release	
ORF9b	Interacts with host protein TOM70 [30, 35]	Inhibit type I interferon [35]
ORF10	Unknown; suspected membrane protein forming viroporin [36]	

Stages of the replication cycle of SARS-CoV-2 have been rapidly inferred from empirical data and extant knowledge of other betacoronaviruses. The first step in cellular infection by SARS-CoV-2 is the binding of S protein to the host cell surface entry factors such as the membrane associated and soluble ACE2 receptor [38] which may be preceded by weaker binding of the S protein to attachment factors such as heparan sulphate [39]. Other entry factors that facilitate attachment or entry include neuropilin-1 [40, 41], the tyrosine-protein kinase receptor UFO (AXL) [42], CD147 [43], high-density lipoprotein (HDL) scavenger receptor B type 1 (SR-B1) [44], integrins [45, 46], angiotension II receptor 1 (AT1) and vasopressin receptor 2, but their role in natural infection is currently unclear. Proteases such as surface TMPRSS2 and endosomal cathespsin L [46] cleave the S protein to activate SARS-CoV-2 entry by endocytosis and membrane fusion [22]. Within the cell, the virus uncoats to release its genomic RNA into the cytoplasm for translation [47]. The translated pp1a and pp1ab polyproteins are proteolytically cleaved to individual Nsps, many of which form the replicase-transcriptase complex [48, 49]. These complexes are localized within specialized double membrane vesicles (DMV). Within the DMV system, the complex operates to replicate genomic RNA and transcribe subgenomic RNAs, which are subsequently translated into structural proteins [50]. Viral assembly occurs within the endoplasmic reticulum, Golgi, and intermediate complex (ERGIC) where membranes studded with viral structural proteins interact with N-encapsidated viral genomic RNA [51]. Pre-activation of S protein by host furin protease may occur before mature viruses are released from the cell by exocytosis of secretory vesicles. As in other coronaviruses, subgenomic RNAs are produced, and most subgenomic RNA consists of a leader sequence in the 5’ untranslated region of the genome and connected to the S gene or other genes in the 3’ end [50]. Hence, translated viral proteins are more abundant towards the 3’ end of the genome, which may affect the sensitivity of diagnostic assays using them as targets for RT–PCR or antigen detection. One exception is the nsp1, which has been shown to be highly expressed and was found to be a sensitive target for RT–PCR [52]. Direct RNA sequencing also reveals the presence of non-canonical subgenomic RNAs in which the 5’ breakpoint is located within the ORF1a gene [50]. As for the putative structural RNA found in SARS-CoV-2, the 5′ UTR has several stem-loops (SL1–5) which may be involved in mediating viral replication as in other betacoronaviruses. The ORF1a-ORF1b junction has a pseudoknotted structure pivotal for programmed ribosomal frameshifting and translation of the ORF1ab polyprotein. The 3′ UTR has the s2 m motif, conserved octanucleotide and many unexpected folds [53].

## Virus evolution

The origin of SARS-CoV-2 is still unknown. Recombination is a frequent event for the viral subgenus *Sarbecovirus*, which contains SARS-CoV, bat SARS related CoV, and SARS-CoV-2 [54]. Some studies suggested that the bat SARS-CoV-2-like coronaviruses are recombinants of lineages related to SARS-CoV and SARS-CoV-2, and SARS-CoV-2 may result from recombinations between these bat SARS related coronavirus and the pangolin SARS related coronavirus [55, 56]. However, another study suggested that recombination may not be involved in the generation of SARS-CoV-2, but the RBD of SARS-CoV-2 shares the same ancestral trait as bat viruses [57]. The divergence date between SARS-CoV-2 and bat sarbecovirus has been estimated to be 1948 [57].

Since its first detection in humans in December 2019, many mutations have been found throughout the SARS-CoV-2 genome [58]. The mutation rate has been estimated to be 1.1 × 10^−3^ nucleotide substitutions per site per year [59]. The time of origin of SARS-CoV-2 was estimated to be late November 2019 [59]. The mutation rate is fastest at the S, N, ORF1ab, ORF3a, and ORF8 genes [60, 61].

In addition to inter-host genetic diversity, mixed viral populations can be present within an individual patient. A variant initially present at low frequency in an individual can become the predominant viral population during the course of illness. In our previous study, we demonstrated the emergence of the S protein W152L mutation in a patient with severe disease [62]. In a study analysing the viral genomes from patients in Austria, an intra-host minor variant was found to be transmitted to others, and become the predominant viral variant in another patient [61].

### Virus mutations and variants

**Figure 2.**
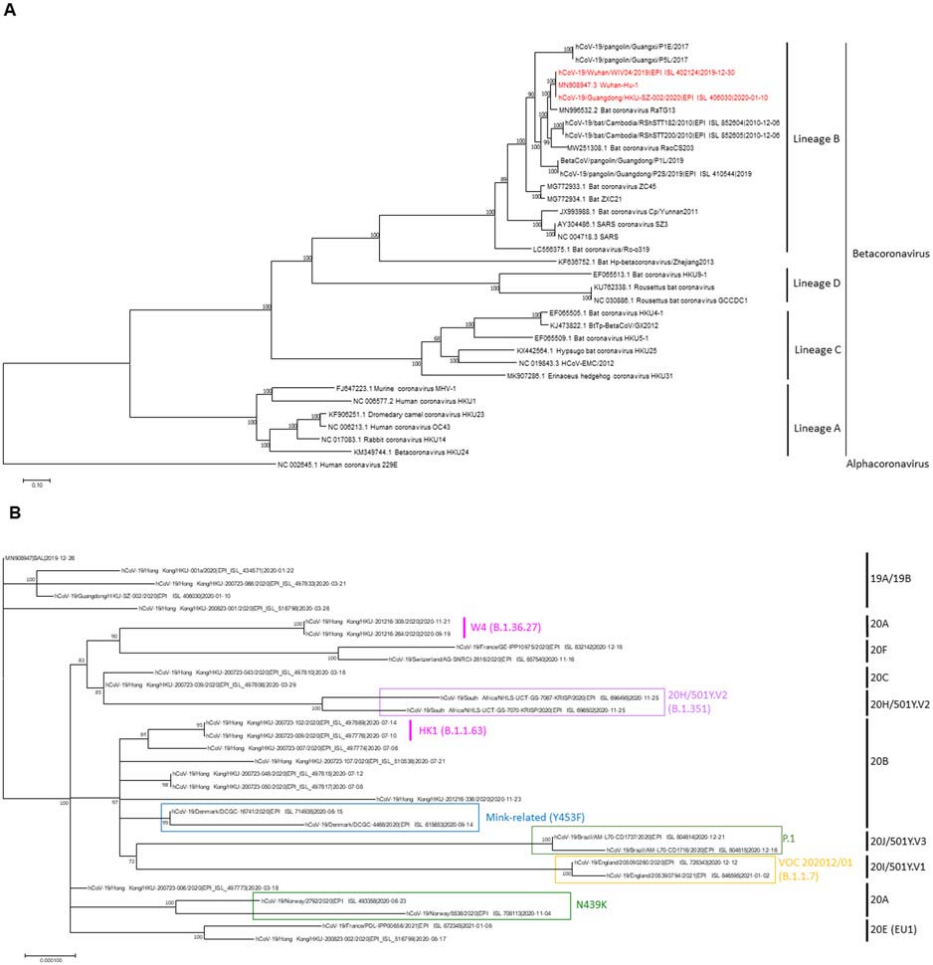
(A) Whole genome phylogenetic tree of betacoronaviruses. The tree was constructed by maximum likelihood method with the best-fit substitution model GTR + F+R5 using IQTree2. Bootstrap values were calculated by 500 trees. SARS-CoV-2 are highlighted in red. Human coronavirus 229E (NC_002645) was used as outgroup. (B) Whole genome phylogenetic analysis showing different clades of SARS-CoV-2. The tree was constructed by maximum likelihood method with the best-fit substitution model TIM2+F + I using IQTree2. Bootstrap values were calculated by 500 trees. Clade information as inferred by Nextstrain or Pango lineage are shown. HK1 is the predominant lineage found during the 2020 summer peak in Hong Kong, while W4 is the predominant lineage that is found in almost all local cases in Hong Kong since November 2020. The reference genome Wuhan-Hu-1 (GenBank accession number MN908947.3) is used as the root of the tree.

SARS-CoV-2 has evolved into different clades and lineages (Figure 2). Currently, there are three major nomenclature systems for the different clades or lineages. The GISAID and Nextstrain systems were used since the beginning of the pandemic, and the clades or lineages are defined by signature mutations. The GISAID clade is currently divided into S, L, and V, and different clades carrying the D614G mutation (G, GH, GR, GV), and O. The Nextstrain is divided into 19 (A, B) and 20 (A-J) according to the year and order when the clade emerged. Although GISAID and Nextstrain nomenclatures are useful in understanding the virus evolution in a macroscopic scale, these systems are not able to delineate more detailed outbreak cluster information. The Pango lineage, first proposed in July 2020, is a dynamic system, which takes into account whether the lineage is actively spreading or not [63, 64]. The Pango lineage system has a much finer resolution than GISAID or Nextstrain, and is particularly useful to capture the emergence of novel variants.

As the virus evolves, many novel variants have been found. The analysis of viral variants helps epidemiological investigations. For example, whole viral genome analysis during the 2020 summer outbreak in Hong Kong showed that the outbreak was most likely linked to viral variants imported by travellers [65]. Furthermore, variant analysis can be used to identify factors that affect transmissibility or virulence of the virus. *In vitro* screening using serial passage or site-directed mutagenesis identified mutations in the S protein that allow the virus to escape neutralization by convalescent plasma or infect cells more efficiently [66, 67].

Long-term SARS-CoV-2 shedding in immunocompromised individuals with acquired hypogammaglobulinemia can lead to a long duration of virus shedding and a larger genetic diversity with continuous turnover of dominant viral species throughout the course of infection. Deletion H69 and V70 in the S protein NTD was reported in a B-cell depleted patient which may be related to selection by convalescent plasma therapy [68]. Furthermore, remdesivir failure with D484Y mutation at RNA dependent RNA-polymerase was also reported in a B-cell immunodeficient patient with protracted SARS-CoV-2 shedding [69].

Viral variants can also emerge during circulation in animals. Since SARS-CoV-2 can infect many animals naturally or experimentally [70], there is always a danger of human-to-animal SARS-CoV-2 transmission, followed by the genesis of mutants in animals which then jump back into human. Mink-to-human transmission has been documented in Europe [71]. Furthermore, the S N501Y variant can be selected in virus adaptation experiments using Balb/c mice [72].

Several notable variants have emerged since the beginning of the COVID-19 pandemic, including B.1.1.7 (VOC-202012/01), B.1.351 (501Y.V2) and P.1 (VOC202101/02) which were first reported from the United Kingdom, South Africa and Brazil, respectively [73]. These variants usually increase transmissibility and, thereby, rapidly replace existing lineages. Some of these variants share certain critical mutations in the S protein RBD. For example, N501Y is present in the B.1.1.7, B.1.351, and P.1, while E484 K is present in the B.1.351 and P.1 in addition to N501Y and D614G (Table 2).

**Table 2.** Amino acid mutations and nucleotide deletions present in each variant.

Variant	United Kingdom (VOC-202012/01)	South Africa (501Y.V2)	Brazil (VOC202101/02)
Pangolin lineage	B.1.1.7	B.1.351	B.1.1.28.1 (Lineage *P*.1)
Number of countries reported with variant^a^	93	45	15
Genes			
orf1ab	T1001I A1708D I2230T	K1655N	S1118L K1795Q
	Del:11288:9 Del:21765:6 Del:21991:3		Del:11288-9
S	N501Y A570D P681H T716I S982A D1118H	D80A D215G K417N E484K N501Y A701V	L18F T20N P26S D138Y R190S K417T E484K N501Y H655Y T1027I
Orf3a			G174C
Orf8	Q27* R52I Y73C		E92K
E		P71L	
N	D3L S235F	T205I	P80R

* stop codon.

^a^ According to the PANGO lineages website https://cov-lineages.org/global_report.html on 21st February 2021.

One of the first major variants identified for SARS-CoV-2 were deletions at the S protein S1/S2 junction. These were readily seen during passage in Vero E6 cells [21]. S1/S2 junction deletion variants have been found to be less virulent in a hamster model [21]. S1/S2 junction deleted variants also naturally exist in patients’ samples before any passage in cell cultures [74].

ORF8 is a unique protein in SARS-CoV-2 [16] and is found to be immunogenic [75]. However, ORF8-deleted or truncated mutants have been identified frequently. In a Singapore study, patients infected with ORF8-deleted mutants have milder disease than those infected with wild-type SARS-CoV-2 [31]. ORF3b deleted mutants have also emerged with the D614G mutation. Truncation of ORF3b confers the loss of its function of interferon antagonism [32].

D614G mutation was not reported in the initial outbreak in China, but is now found in almost all strains globally. Several studies have evaluated the impact of D614G on the SARS-CoV-2. Collectively, they show that D614G variant replicates to a higher titre *in vitro* and *in vivo*, and transmits more efficiently, but does not affect disease severity or confer a significant change in neutralizing activity of convalescent sera [66, 76–78]. Mechanistically, D614 mutation affects the conformation of the S protein, which allows more efficient binding to the human ACE2 receptor [79].

There have been multiple outbreaks of SARS-CoV-2 infection among mink farms in Europe [80]. Mink-associated human infections have been identified [71]. A unique lineage has been found in these mink-associated human cases from Denmark, including 4 mutations in the S protein (Δ69-70, Y453F, I692 V, M1229I). Y453 has been shown to be involved in receptor binding [66]. Preliminary investigation with 9 COVID-19 convalescent serum specimens showed a statistically significant reduction in neutralizing antibody titre [73].

The B.1.1.7 variant was first detected in September 2020, spread rapidly in south-eastern England by December, and has become the predominant variant in the UK. This variant has increased transmissibility and is now found worldwide [81]. This variant is defined by 17 mutations, including a non-synonymous S N501Y at RBD, and the P681H mutation which is located in the furin cleavage site. However, no change in neutralizing activity by sera of vaccine recipients of the BNT162b2 mRNA vaccine was found against pseudoviruses bearing the Wuhan reference strain and the B.1.1.7 variant [82].

The B.1.351 variant has rapidly increased in South Africa in late 2020. This variant possesses several mutations in S protein NTD (L18F, D80A, D215G, Δ242-244, R246I) and RBD (K417N, E484 K, and N501Y). Monoclonal antibody or nanobody targeting the S protein amino acid positions 417 or 484 showed reduced binding to the B.1.351 variant [83]. Furthermore, neutralizing antibody against the B.1.351 variant could not be detected in 48% of convalescent sera of COVID-19 patients [83].

The P.1 variant has 17 unique mutations including RBD E484 K and N501Y mutations has emerged [84]. This is a descendent of the lineage B.1.1.28.1, and now known as the P.1 lineage which is mainly limited to Brazil, but has also been reported in Japan, Korea, and Faroe Islands [85].

A key concern about viral variants is whether they increase the risk of reinfection or vaccine failures. In the first case of reinfection reported in August 2020, the second episode was caused by a D614G variant [86]. In a reinfection case reported from Brazil, the second episode was caused by the E484 K variant [87]. Virus carrying the E484 K was shown to be less susceptible to neutralization by sera from mRNA vaccine recipients [88].

## Transmission routes

SARS-CoV-2 is believed to spread predominantly via short-range airborne aerosol, respiratory droplets, and direct or indirect contact with infectious respiratory droplets. Airborne transmission of SARS-CoV-2 has been elegantly demonstrated in the hamster model [89, 90]. Low level of SARS-CoV-2 RNA (concentrations in air up to 3.4 × 10^3^ RNA copies per m^3^ air sampled) could be detected in the air samples obtained from the environment housing COVID-19 patients even in the absence of aerosol-generating procedures [91–94]. Viable SARS-CoV-2 virus could be isolated from air samples collected as far as 4.8 m away from COVID-19 patients with estimated viral concentrations of 6–74 TCID_50_ units/L of air [95], substantiating the hypothesis that aerosol dissemination of SARS-CoV-2 may serve as a source of infection. Large quantities of particles, with the majority of less than 5 microns, can be emitted during normal speech, and the amount is positively correlated with the loudness of vocalization [96]. Aerobiological study showed that particles produced in the human respiratory tract represent a continuum of sizes instead of a sharp distinction into respiratory droplet (≥5 microns) or airborne aerosol (<5 microns). The concentration of respiratory droplets and airborne aerosol carrying SARS-CoV-2 should be inversely proportional to distance from the source patient. Short-range airborne spread should be the predominant route of SARS-CoV-2 transmission.

In addition, contact with frequently touched surfaces, shared items, and food that are contaminated by infectious respiratory droplets likely represent another route of transmission of SARS-CoV-2 [97]. One study found that 5% of the near-patient environmental samples contained SARS-CoV-2 RNA with a median viral load of 9.2 × 10^2^ copies/mL [98], with the highest contamination rates on patients’ mobile phones, floors, bed rails and air exhaust vents [91–93, 98]. Though still considered controversial as a portal of transmission, several outbreaks have been linked to contaminated frozen food, their packaging materials and storage environments [99]. The half-life of SARS-CoV-2 infectivity was 1.7–2.7 days at 20°C, which is reduced to a few hours at 40°C [100]. At the highest viral load excreted by infectious patients, viral particles remained viable for up to 28 days at 20°C on common surfaces such as glass, stainless steel, and polymer banknotes [100]. The relative humidity also affects the rate of viral decay, which was most rapid at 65% relative humidity and slower either at lower (40%) or higher (75%) humidity [101].

Other routes of transmission, including faecal–oral, and contact with various body fluids including urine, tears, and breast milk, have been postulated [102–106]. Indeed, oral SARS-CoV-2 inoculation can establish subclinical respiratory infection with virus shedding in the hamster model [107]. Human vertical or perinatal transmission from mother to babies is rare but possible [108].

Vertical transmissions in high rise buildings by faecal aerosols through chimney effect, wake effect and minor leaks in sewage, vent pipes, or light wells were reported [109]. However, the significance of these alternative routes of transmission in driving the community epidemic is still unclear.

## Epidemiological characteristics

The mean incubation period of SARS-CoV-2 infection was 4.0–5.2 days, and incubation period of longer than 14 days has been reported [3, 110]. During the early stage of the pandemic, the mean serial interval was 4.0–7.5 days [3, 110, 111], the epidemic doubling time was 6.5–7.4 days [3, 112], and the highly context-dependent basic reproductive number (*R*_0_) was 2.2–2.7 [3, 113, 114]. But estimating *R*_0_ with precision is difficult due to the substantial proportion of undetected cases and varying testing policies. Literature on transmission heterogeneity is scarce. Heterogeneity in infectious disease dynamics, where most individuals infect only a few others while a small subset of the population is responsible for the majority of new cases, is commonplace. Retrospective history from 135 cases between 21 January and 26 February 2020 in Tianjin, China, showed significant transmission heterogeneity with a coefficient of dispersion of 0.25 [115]. The estimated overall infection fatality ratio (IFR) in China was 0.66% which increased with age [116]. This is similar to the IFR estimate of 0.6% inferred using the corrected IFR on the Diamond Princess cruise ship [117].

An important reason for the rapid spread of COVID-19 is the presence of asymptomatic and presymptomatic transmission. Asymptomatic or mildly symptomatic cases constitute 30–60% of all patients infected with SARS-CoV-2 [118, 119]. In *ex vivo* human lung tissues, SARS-CoV-2 generated 3.2-fold more infectious virus particles than did SARS-CoV-1, but did not significantly induce host pro-inflammatory response [120], which explains the high proportion of asymptomatic or mildly symptomatic cases in the COVID-19 pandemic. Moreover, in contrast to SARS-CoV-1 patients whose viral load in nasopharyngeal aspirates peaked at around day 10 of symptoms [121], the viral load in the respiratory samples of COVID-19 patients was highest during the first few days of symptom onset [90]. It was estimated that presymptomatic transmission accounted for 4.2–44.4% of secondary COVID-19 cases [122–125]. The secondary attack rate within Wuhan households was 15.6%, with the presympomatic cases being the most infectious [126]. In addition, the lack of herd immunity at the early stage of the pandemic adds to the susceptibility of the general population. The estimated seroprevalence rate in Wuhan was 3.2%−3.9% in March 2020 [127–129], and similar figure of 4.1% was recorded in California in April 2020 [130].

Mask-off activities such as dining, singing, swimming, and other physical activities are especially dangerous in overcrowded indoor venues with suboptimal ventilation or contaminated frequently-touched surfaces that are poorly sanitized [131]. Thus outbreaks have been reported as clusters in family homes, restaurants, bars, markets, religious premises, cruises, carriers, construction sites, dancing studio, schools, nursing homes, and healthcare facilities [132]. Several superspreading events have been highlighted. A British individual who attended a conference in Singapore in January 2020 has spread the virus across the UK, France, and Japan through the exposure at a ski resort, where 13 of the 21 exposed people eventually tested positive [133]. From late February to early March 2020, an outbreak associated with the Sunday worshipping event in a church caused 61.3% of the 8162 confirmed COVID-19 in the Republic of Korea [134, 135]. The outbreak related to an index patient on the Diamond Princess cruise ship has led to the quarantine of the passengers and cruise members at the Port of Yokohama in Japan, on which 696 of the 3711 passengers (18.8%) tested positive for SARS-CoV-2 [118]. In fact, using a susceptible–exposed–infectious–removed (SEIR) model that integrates dynamic mobility networks based on mobile phone data, a small minority of “superspreader” at points of interest, most notably full-service restaurants, was found to account for a large majority of COVID-19 cases [136]. Selective implementation of specific restrictive measures at these critical control points of interest may be most effective. Hospital outbreaks at wards, dialysis centres, and outpatient clinics [137, 138] fuel the community outbreaks and vice versa which adds to the burden of infection control.

The long environmental survival of SARS-CoV-2, high proportion of asymptomatic or mildly symptomatic patients, peaking of viral load before or at presentation and therefore its high transmissibility warrants universal masking, diligent hand hygiene, and stringent social distancing measures for the successful control before the herd immunity is built up by vaccination.

## Histopathology and pathogenesis of COVID-19

**Figure 3.**
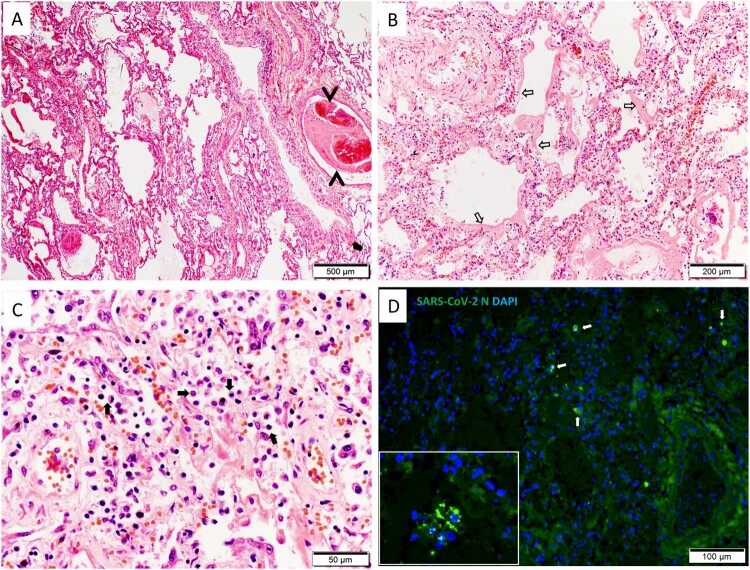
Histology of lung tissue section. (A) Image of hematoxylin and eosin (H&E) stained lung tissue shows diffuse alveolar exudation and inflammatory infiltration; a medium size blood vessel containing thrombus which almost blocks the entire lumen (arrow heads). Scale bar = 500 µm. (B) Magnified H&E image shows severe hyaline membrane formation in the alveolar space (open arrows). Scale bar = 200 µm. (C) Magnified H&E image shows severe mononuclear immune cell infiltration in the alveolar space (solid arrows). Scale bar = 50 µm. (D) Immunofluorescence stained SARS-CoV-2 nucleocapsid (N) antigen in alveoli (white arrows); the insert image showing a few N protein expressing cells in a small bronchial lumen. Scale bar = 100 µm.

SARS-CoV-2 can cause infection in multiple organs as shown in both *in vitro* and *in vivo* studies [139–150]. with common histopathological features summarized in Table 3. Autopsy showed that pulmonary involvement with diffuse alveolar damage together with hyaline membrane formation and pulmonary micro-emboli are the most prominent acute histopathological findings [151] (Figure 3). These features were often associated with high inflammatory cytokines and increased angiogenesis in fatal cases [152, 153]. The hyaline membrane was attributed to an increase in vascular permeability (termed as “bradykinin storm”) and accumulation of hyaluronic acid in the alveolar space, leading to trapping of high volume of water [154]. Moreover, serum autoantibodies directed against many immunomodulatory proteins including cytokines, chemokines, complement activation components, and cell surface proteins were found in a high throughput extracellular antigen profiling study which may add to the tissue damage by immune complex deposition and complement [155]. These autoantibodies may also impair immune function and virological control by inhibiting immunoreceptor signalling. The presence of these autoantibodies including those against interferons is strongly associated with disease severity [156].

**Table 3.** Histopathology and pathogenesis of COVID-19.

Organ	Histopathology	Features of vascular involvement	References
Lung	Diffuse alveolar damage with lymphocytic/ monocytic infiltrate together with intra-alveolar fibrinous exudate, hyaline membrane formation at acute stage. Type II pneumocyte hyperplasia with interstitial fibrosis at late stage Increase in pulmonary megakaryocytes	Perivascular cuffing by lymphocytes with fibrin/ hyaline thrombi seen within pulmonary vessels and capillaries Congested vessels	[143, 151, 153]
Heart	Small or multifocal lymphocytic infiltrate with dysmorphic cardiomyocyte and rare necrosis (milder pathology when compared with the lung) Eosinophilic myocarditis (rare)	Epicardial capillaries with prominent lymphomonocytic endotheliitis Macrovascular or microvascular thrombi Intraluminal megakaryocytes	[144, 151]
Brain	Activation of astrocytes and microglia with infiltration of cytotoxic T cell mainly in brainstem and meninges Occasional expression of viral antigen at cortical neurons	Intravascular thrombi with perivascular microhaemorrhages and intramural inflammatory infiltrates Multiple microscopic ischaemic infarct with or without antigen expression at endothelium	[174-179]
Kidney	Acute tubular injury Interstitial fibrosis Podocyte vacuolation Loss of brush border in proximal tubule Focal segmental glomerulosclerosis Granulomatous interstitial nephritis	Hemosiderin granules and pigmented casts, together with abundant erythrocyte with obstruction of peritubular capillary lumen with activation of endothelium	[143, 145, 167]
Liver	Histiocytic hyperplasia Focal macrovascular and microvascular steatosis Patchy hepatic necrosis in centrilobular and periportal areas	Platelet fibrin thrombi in sinusoid, central vein or portal vein Megakaryocytes in sinusoid Sinusoidal congestion Ischaemic necrosis	[143, 145, 167]
Spleen	White pulp depletion	Splenic infarction	[143]
Skin	Parakeratosis, acanthosis, dyskeratotic keratinocytes, necrotic keratinocytes, acantholytic clefts, lymphocyte satellitosis and pseudoherpetic of the epidermis	Dermal infiltrate with perivascular and intramural lymphocyte in muscular wall of small vessels Occasional intravascular hyaline/ fibrin thrombi Vascular deposition of C4d by immunohistochemical staining	[145, 162]
Placenta		Villous infarction, atherosis and fibrinoid necrosis of maternal vessels	[146]
Testis	Interstitial edema with leukocyte infiltration Sertoli cells showed swelling, vacuolation and cytoplasmic rarefaction, detachment from tubular basement membranes, and loss and sloughing into lumens of the intratubular cell mass		[147]

Although SARS-CoV-1 is more virulent based on *in vitro* studies in terms of replication and cell damage [142, 148], SARS-CoV-2 appears unique in causing endotheliitis [152, 157], as evident by viral particles in vascular endothelium using electron microscopy [157]. Soluble endothelial markers such as angiopoietin-2 level are positively correlated with severity of COVID-19 [158]. Furthermore, endotheliitis increases propensity of thromboembolism and multisystem involvement in COVID-19 patients [152, 157, 159, 160]. Widespread thrombosis could be related to the hyperinflammatory and hypercoagulopathy states, termed as “immune-thrombosis” [160, 161]. Direct endothelial injury triggers innate immune response, including activation of monocytes and complement pathways, leading to deposition of terminal complement components C5b-9 (membrane attack complex), C4d [162], and mannose binding lectin (MBL)-associated serine protease (MASP) in the microvasculature [163]. Complement and endothelium activation induce the production of von Willebrand factor (vWF) and factor VIII (FVIII), while reducing antithrombin and ADAMTS13 activity [164]. Activated neutrophils release neutrophil extracellular traps to stabilize microthrombi [165–167]. Macro- and micro-vascular thrombosis and intraluminal megakaryocyte are more common features than lymphocytic infiltration of myocardium in patients with cardiac involvement [168]. In terms of lymphoid organ involvement, T-cell depletion occurred in the spleen [169]. Necrosis or atrophy in the lymphoid tissue of lymph nodes and white pulp of the spleen are commonly observed extrapulmonary pathologies [170].

COVID-19 may affect the central nervous system due to indirect effects of cytokine storm or suspected direct virus invasion. The S1 protein can cross the blood brain barrier in a mouse model. Furthermore, intranasally administered S1 also entered the brain with significant uptake at olfactory bulb and hippocampus, although at levels around 10 times lower than that after intravenous administration [171]. The endotheliitis and systemic inflammatory response syndrome with neuronal activation are also postulated to explain the neurological manifestations [172, 173]. Autopsy studies of the brain showed that ischaemic infarct with perivascular microhaemorrhage together with neutrophilic plugs or intravascular microthrombi were common features [174–179]. Similarly, radiological features of vascular inflammation were observed in magnetic resonance imaging (MRI) of the brain [180]. T-cell lymphocytic infiltrates were commonly seen at perivascular, parenchymal as well as leptomeningeal areas with microglial and astrocyte activation [167, 169, 175, 178, 179]. Clinical improvement of encephalopathic symptoms with steroid tends to suggest a dominant role of inflammatory response [181]. The detection of SARS-CoV-2 in the brain and cerebrospinal fluid by immunohistochemical staining and RT–PCR yield inconsistent results [167], as viral RNA detected from brain biopsy may come from vascular endothelium instead of neurons. The localization of S antigen and visualization of virus-like particles at the endothelium were observed in some patients with endotheliitis while only few could demonstrate S protein expression in cortical astrocytes [174, 182]. The more consistent finding is the expression of virus nucleocapsid antigen in olfactory sustentacular and horizontal basal cells in some patients and also in the olfactory neurons in infected hamsters, suggesting that direct neuronal invasion by virus is possible [183].

## Immunological profile of patients with COVID-19

Innate immunity is the first line of defence against infection. Yet SARS-CoV-2 may evade innate immunity by antagonizing host interferon response. Viral proteins that have been shown to antagonize interferon response include Nsp1, Nsp3, Nsp12, Nsp13, Nsp14, Nsp15, ORF3, and ORF6 [27, 142, 184–186]. Furthermore, the frequency of dendritic cells, T cells, NK cells, and monocytes was significantly reduced in the peripheral blood of acute patients when compared with healthy donors [187]. In particular, decrease in levels of CCR2 expression in dendritic cells may lead to poor maturation on stimulation [188], further reducing levels of interferons, and hence poor stimulation of CD4+ and CD8+ T lymphocytes during acute phase of infection [187]. Furthermore, infiltration of monocytes/macrophages in lungs can increase pro-inflammatory cytokines and chemokines such as IL-6 and IP-10, which fuels the cytokine storm [189]. Th2 cytokines, such as IL-5 and IL-13, are elevated in patients with severe COVID-19 [190].

For humoral adaptive immune response, most recovered patients develop SARS-CoV-2-specific IgA, IgG, and IgM response not only against S (including RBD) and N but also other non-structural proteins [191–193]. The peak antibody response appears at around 1 month [194] and is higher among patients with more severe disease [195]. Most studies showed a static or slow decline in neutralizing antibody and IgG response after few months [196, 197], while IgA and IgM declines more rapidly [194]. One study estimated the half-life of S protein IgG to be 140 days [198]. Notably, some patients who recovered rapidly showed increasing titres over time [196]. The IgG and IgM levels in saliva correlate with those of serum [194]. There is also a difference in antibody response between adults and children. While adults develop antibody against both N and S proteins equally well, children have a stronger anti-S antibody response than anti-N antibody [199]. S-specific memory B lymphocytes showed increase in abundance over time, suggesting that patients can develop rapid antibody response during reinfection, as was seen in our previously reported reinfection case [198, 200, 201]. The exact duration of detectable serum neutralizing antibody titre after natural infection or vaccination still awaits long-term follow-up study.

Cases of reinfection have been reported [86], and neutralizing antibody could not be detected at presentation of the second episode of infection 5 months after the first episode [201]. Magnitude and duration of persistence of IgG or neutralizing antibody correlate with severity of COVID-19 in some studies [193, 195].

For cell-mediated adaptive immunity, SARS-CoV-2 leads to T-cell lymphopenia and functional impairment of both CD4+ and CD8+ T cells during the acute stage [187, 202]. Total CD4+ and CD8+ T cells are reduced in both mild and severe diseases, but particularly lower among severe cases [203]. SARS-CoV-2 specific CD4+ and CD8+ T cells can be detected in about 50% of patients during the acute period and >80% of patients in the convalescent stage [204, 205]. The development of SARS-CoV-2 specific T-cell response is impaired among patients with severe COVID-19 [204]. There is a higher frequency of memory CD4 than CD8 T-cell responses against N and RBD [204]. SARS-CoV-2-specific CD8+ memory T-cell responses are directed primarily to the S and M proteins especially among those who recovered from severe COVID-19. The levels of T_H_17 cells were elevated among severe cases [206]. The frequency of T follicular helper cells during the convalescent phase is higher among patients with severe disease than those with milder disease, which correlated with the neutralizing antibody titre [207]. SARS-CoV-2 specific T-cell immunity can also be found in up to 83% of non-COVID-19 individuals which may suggest some cross reactive T-cell immunity that may or may not be protective [208–210]. Pre-existing memory CD4+ T cells are cross reactive for SARS-CoV-2 and other seasonal coronaviruses [210]. SARS-CoV-2-specific T lymphocytes (CD4+, CD8+) decreased with half-lives of 3–5 months [198].

The overall clinical phenotype of COVID-19 is determined by the degree of early control of viral load by innate and adaptive immune responses, the inflammatory and apoptotic damage of cells triggered by the burden of virus, the functional reserve of the affected organs and the compensatory regenerative or reparative power of the host tissues.

## Clinical manifestations

COVID-19 is primarily a respiratory disease which can manifest as acute upper or/and lower respiratory tract syndrome of varying severity. The symptom onset of COVID-19 is more likely to be gradual than the abrupt onset in influenza. The patient can manifest with asymptomatic virus shedding, or a self-limited syndrome of fever, fatigue, myalgia, arthralgia, rhinorrhoea, sore throat, and/or conjunctivitis at one end of the spectrum. But it can also progress to persistent fever, cough, hemoptysis, silent hypoxia, chest discomfort or pain, respiratory failure, or even multiorgan failure [211, 212]. Impairment of smell (hyposmia, anosmia, and parosmia) or taste (dysgeusia) has been recognized as important chemosensory disturbances in COVID-19 [213]. Non-conductive olfactory dysfunction (OD) may be the sole manifestation [214]. Other extrapulmonary manifestations include diarrhoea, lymphopenia, thrombocytopenia, deranged liver and renal function, rhabdomyolysis, meningoencephalitis, stroke, seizure, Guillain–Barré syndrome, cardiac arrhythmia or heart block, pancreatitis, Kawasaki disease like multisystem vasculitis, skin rash or chilblain-like lesions, thromboembolism, and acute thyroiditis [215–217]. In an analysis of 72314 COVID-19 in China up to 11 February 2020, 81% of the laboratory confirmed patients had mild to moderate illness, 14% had severe disease, and 5% were critically ill requiring intensive care [218].

**Figure 4.**
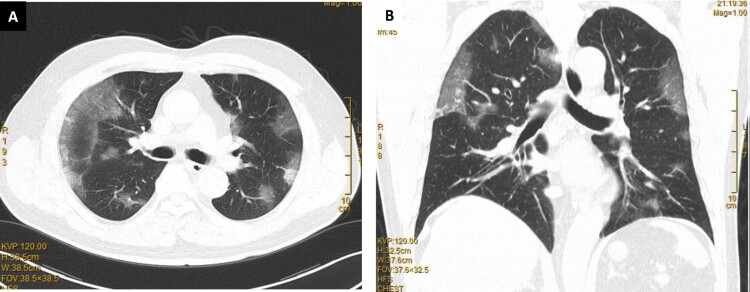
Typical changes of COVID-19 pneumonia on lung computed tomography showing bilateral multifocal patchy ground glass opacities: (A) transverse view; (B) coronal view.

Clinical improvement of mild and moderate cases generally occurs around 10 days after symptom onset which coincides with at least 1 log reduction of respiratory viral load [219] and the rise of serum antibodies against N or S protein [220]. However, clinical deterioration of moderate disease to respiratory failure may also occur at this time with persistent salivary viral load and increasing lymphopenia in these worsening patients [221, 222]. Chest radiograph or lung CT scan typically showed bilateral multifocal and peripheral ground glass opacities (Figure 4) which may deteriorate to dense consolidation in progressive disease [223]. The radiological abnormalities usually peak by 2 weeks after symptom onset and are replaced by fibrosis with recovery [224]. The prognosis of COVID-19 is worse in elderly obese males or those with comorbidities such as hypertension, diabetes mellitus, atherosclerotic vascular diseases, vitamin D deficiency, and other chronic medical illness [225]. Patients with X-linked putative TLR7 loss of function variant, autoantibody against type 1 interferons, defective mutations of IFNAR2 or other interferon signalling genes, antiviral restriction enzyme activators (OAS), blood group A and the associated SNPs found by GWAS are associated with severe disease [226–230]. Acute kidney injury affected >20% of hospitalized patients and >50% of those requiring ICU admission, but the rate varies widely between studies [231]. The overall crude fatality rate for laboratory confirmed cases is about 2% [232], but can be as high as 21.9% in patients over 80 years of age [233]. Early bacterial and fungal superinfections are uncommon but late superinfections, including invasive pulmonary aspergillosis, were reported in those with prolonged ICU stays and treatment by immunomodulatory agents [234].

Follow-up study at 6 months after COVID-19 symptom onset showed that over 60% of these patients had persistent symptom of fatigue or muscle weakness [235]. Sleep difficulties (26%), anxiety or depression (23%) were not uncommon [235]. Other symptoms include smell or taste disorder, palpitations, joint pain, dizziness, diarrhoea, vomiting, and chest pain which constitute a constellation of symptoms termed “post-acute COVID-19 syndrome”. This group of patients are also called “COVID long haulers” [236]. Those with severe disease requiring respiratory support had lung diffusion impairment [235]. However, little objective evidence of post-acute COVID-19 syndrome can be found on investigations which bear some similarity to chronic fatigue syndrome or myalgic encephalomyelitis. The cause was speculated to a dysregulated immune system which was activated to fight SARS-CoV-2 but failed to dampen down afterwards [237]. The other differential diagnoses are either an autoimmune process triggered by SARS-CoV-2 or a persistent SARS-CoV-2 infection which cannot be easily detected by conventional testing [238]. The relationship between the presence of serum autoantibodies and the post-acute COVID-19 syndrome requires further investigations.

In general, children have a shorter and milder disease than adults [239, 240]. However, a rare but life-threatening Kawasaki-like disease, known as multisystem inflammatory syndrome in children (MIS-C) or paediatric inflammatory syndrome temporally associated with SARS-CoV-2, are seen during the convalescent phase of the illness [97, 241]. Children with MIS-C are usually older, has a lower lymphocyte and platelet count, and a higher level of CRP and ferritin than those with Kawasaki disease [242].

## Laboratory diagnosis

One of the most important aspects in curbing the spread of the virus and improving the prognosis is rapid yet accurate diagnosis of infection followed by timely isolation, contact tracing and treatment. Molecular testing is now the mainstay of diagnosis, supplemented by point-of- care antigen testing (POCT) [243]. Antibody detection aids in assessment of immunity, contact tracing, and disease prevalence in the population. A multitude of diagnostic platforms, both in-house and on commercial platforms, are developed to meet these demands [244].

### Specimen collection

Viral load in the respiratory tract is highest at or soon after symptom onset [222], and it decreases at a rate of 1 log_10_ per week [90]. Testing nasopharyngeal aspirate, nasopharyngeal swab, or throat swab is adequate for early-stage infection, especially asymptomatic or mild upper respiratory tract infections. Patients with lower respiratory tract symptoms should send sputum to enhance sensitivity [245]. Though broncho-alveolar lavage (BAL) showed the highest positive rate among different respiratory specimens, it is only indicated in those with severe lower respiratory tract involvement when the nasopharyngeal and throat specimens are tested negative [246]. Posterior oropharyngeal secretion (POS) or deep throat saliva is increasingly studied as it represents a pooling of posterior nasopharyngeal, oropharyngeal and lower respiratory secretions during the supine position during sleep, when taken in the early morning before breakfast and mouth rinsing [90, 247]. It can be self-collected by patients with instructions, reducing patient discomfort, circumventing swab shortage, and minimizing aerosol exposure for health care professionals. The cost of collecting POS could be 2.59-fold lower than nasopharyngeal specimen [248]. The sensitivity is comparable with nasopharyngeal swab in properly collected specimens by cooperative patients [249–252] . The sensitivity does not vary much between early morning and at least 2 h after meal [253].

For non-airway specimens, viral shedding by RT–PCR was found in faecal material in 40.5% of patients after the first week of symptom onset and could persist for 3 weeks or more [254]. Presence of viral RNA in the sewage system may provide a cost-effective and non-invasive way of monitoring the disease spread within the community and may serve as an early warning system for population that lacks access to healthcare [255]. Enveloped virus has affinity to biosolids which may allow testing of sludge at sewage treatment plants with better sensitivity than testing influent [256]. Composite sampling is used in most studies [257]. Sewage sample should be concentrated followed by efficient RNA extraction to prevent inhibition of molecular assays [256]. Viral RNA can also be detected in the blood in about 30% of severe patients, but the detection rate is much lower in milder cases [90, 246]. Even without ocular symptoms, the conjunctival secretion may contain a small amount of SARS-CoV-2 RNA in around 8% of patients [258]. Viral RNA is rarely found in the urine [246].

To accommodate the large amount of specimen for screening asymptomatic population, pooling of clinical specimens, up to 5–30 samples per pool [259, 260], is an additional strategy to cope with reagent shortages, at the expense of possibly longer processing time and reduced diagnostic sensitivity of the weakly positive sample [261]. It is efficient only when the expected positive sample number is low as positive pool requires individual retesting [262]. Strategic retesting of a defined group [263] and the use of mathematical models to stratify pool size by age groups based on their respective disease prevalence may improve efficiency [264].

### Molecular testing

Reverse-transcriptase polymerase chain reaction (RT–PCR) is the most widely used technique. Potential molecular targets for SARS-CoV-2 include structural proteins (e.g. S, E, helicase (hel), N, M and non-structural regions such as the RNA-dependent RNA polymerase region (Rdrp), and other ORF1ab targets [52, 265, 266]. There is currently no consensus on which gene confers the best diagnostic performance. Presently, one bat SARS related CoV conserved and one SARS-CoV-2 specific target regions are recommended to mitigate effect of random mutation or genetic drift while maintaining specificity [265]. However, mutations can affect the sensitivity of detection by RT–PCR. For example, mutations in the S gene of the UK variant B.1.1.7 has led to the failure of some RT–PCR primers targeting the S gene [267].

High throughput and automated commercial platforms have been developed for molecular SARS-CoV-2 diagnosis. Molecular POCT enables rapid testing near the site of collection in areas with little laboratory support [249]. To improve diagnostic sensitivities of molecular assays, clustered regularly interspaced short palindromic repeat (CRISPR)-based technology has been employed by coupling with Cas enzyme [268]. Target enrichment sequencing by NGS with nanopore or Illumina technology can unravel the entire genome within a few days. Sharing of genetic data facilitates tracking of disease spread, understanding of disease transmission route, monitoring of viral genome evolution and detecting novel variants.

### Antigen detection

N is abundantly expressed in SARS-CoV-2 and is thus widely used as the target for COVID-19 antigen test [269]. Detection is achieved by capturing viral antigen in clinical specimens by monoclonal antibodies fixed on a membrane in colorimetric lateral immunoassays. Though this assay can be delivered as POCT in an outpatient or even non-healthcare setting, it has low sensitivity when compared with RT–PCR assays especially for samples with low viral load. In general, antigen test is negative when their Ct values on quantitative RT–PCR are more than 25, although the Ct values vary with different assays and conditions [270].

### Antibody detection

While antibody testing is generally not useful for acute management, it can be used for retrospective diagnosis and seroprevalence study to understand herd immunity [271]. Commonly employed techniques are lateral flow, chemiluminescent, immunofluorescent, and enzyme-linked immunosorbent assays [272, 273]. Median seroconversion times following symptom onset are 11 days for total antibodies, 12 and 14 days for IgM and IgG respectively [220]. After 14 days, 56–97% of patients develop IgM and 91–100% of patients develop IgG [274], with no significant time difference between IgM and IgG response [275]. IgM peaks at around 3 weeks after symptom onset and falls to baseline level after day 36 [276]. The duration of IgG or neutralizing antibody positivity remains controversial. Some study showed decrease in neutralizing antibody titre within 3 months after symptom onset, while others showed no such decrease [200]. Antibody development against S and N protein is comparable by 1 month after infection [277]. Titre of anti-S or anti-S RBD antibody may better reflect protection against reinfection [277].

Traditional neutralization assay requires manipulation of live virus and necessitate biosafety level 3 laboratories. Pseudovirus neutralization assay using vesicular stomatitis virus (VSV) expressing S protein of SARS-CoV-2 containing the RBD, can be used in biosafety level 2 facilities [278]. Neutralizing antibodies are directed towards the RBD and NTD. Both sites are situated at the tip of the S protein. Surrogate virus neutralization assay based on antibody-mediated blockage of RBD-ACE2 interaction has been developed [279].

Studies have shown serological cross-reactivity between SARS-CoV-2 and SARS-CoV, with decreasing frequency of cross-reaction from N protein, S protein to RBD domain by enzyme immunoassay [52, 280], with no significant cross neutralization [281]. Cross-reactivity against other seasonal human coronaviruses in SARS-CoV-2 infection has been shown as well, though intensity is not as great as that with SARS-CoV [52, 281, 282].

Antibody test has also been used to assess whether SARS-CoV-2 has circulated in the population before the isolation of the virus. A study from Italy reported that anti-RBD antibody could be found in blood samples collected as early as September 2019 [283]. In the United States, 106 of 7389 of residual specimens from blood donors collected between 13 December 2019 and 17 January 2020, tested positive for IgG against SARS-CoV-2, and neutralizing antibody was detected in 84 of 90 of these samples [284]. Though these studies suggest that COVID-19 may have emerged much earlier than the first RT–PCR confirmed case, the possibility of EIA cross-reactivity with other coronaviruses cannot be excluded.

### Viral culture

Infectiousness of SARS-CoV-2 in clinical specimens can only be demonstrated by cell culture assays in biosafety level 3 facilities. Furthermore, cell culture is essential for the evaluation of potential antiviral compounds and vaccines [285]. Viral culture turned negative in 97% of patients by 10 days after symptom onset, coinciding with the time of seroconversion [286]. Duration of live virus shedding is believed to be even shorter in faecal specimen [287]. Shedding is prolonged in severe and immunocompromised cases [288].

Vero E6 cells which have abundant ACE2 expression are commonly used for virus isolation [142]. Vero E6 cell line that expresses TMRPSS2 can result in better culture yield and reduce the likelihood of *in vitro* selection of S1/S2 junction site deletion mutant [289, 290]. SARS-CoV-2 also grows in human continuous cell lines such as Calu3 (lung cancer), Huh7 (liver cancer) and Caco2 (colonic cancer) [142]. It grows modestly on U251 (glioblastoma) which is not seen with SARS-CoV-1 [142]. Organoid systems such as bat and human intestinal organoids are susceptible to SARS-CoV-2 and are developed to better study tissue tropism, the dynamics of infection and testing of therapeutic targets. SARS-CoV-2 is successfully cultured in human intestinal organoids from a stool specimen with high *C*_t_ value of 33.6, demonstrating possible enteric infection by oro-faecal route [141].

## Treatment

Except in places where all infected cases are legally required for mandatory hospital isolation, most patients with mild symptoms require only home isolation, monitoring, and symptomatic treatment. Those with persistent fever, fatigue, and dyspnoea would require admission for full assessment, respiratory support, and targeted anticoagulation by low molecular weight heparin to prevent thromboembolic events. Since the viral load peaks at the time of symptom onset or presentation [90], antiviral treatment is unlikely to work unless given early when the disease is still mild. Remdesivir has been shown to shorten the duration of hospitalization by 5 days in a randomized control trial which did not monitor the serial viral load changes after treatment [291]. The WHO Solidarity trial, a multinational trial with 11,330 adult patients, found that remdesivir, lopinavir-ritonaivir, interferon β-1a, and hydroxychloroquine, have little or no clinical benefit when given as monotherapy, especially when started at the stage of respiratory failure [292]. However, a combination of interferon β-1b, lopinavir-ritonavir and ribavirin was shown to shorten the duration of hospitalization and reduce the viral load by 2–3 log between day 6 and day 11 after symptom onset if given early in a randomized control trial [293]. Similarly, inhaled interferon β-1a was also shown to improve symptoms in mild cases in another randomized control trial without viral load monitoring [294]. This is not unexpected because while SARS-CoV-2 is highly susceptible to interferons *in vitro*, the virus was shown to reduce type 1 interferon produced in *ex vivo* infected lung tissue explant [120, 295]. Furthermore, about 13% of patients with severe COVID-19 were found to have high titres of auto-antibody against type 1 interferons and especially against interferon-α [156].

Though individual or cocktail neutralizing monoclonal antibody treatment has been shown to reduce viral load when given early after symptom onset and before the appearance of serum anti-SARS-CoV-2 antibody in non-hospitalized patients [296, 297], this approach has not yet been shown to reduce morbidity and mortality. A clinical trial of a monoclonal antibody, LY-CoV555, did not show clinical benefit among hospitalized patients [298]. Similarly, convalescent plasma with neutralizing antibody only improved clinical status of elderly with mild COVID-19 when given within 3 days of symptom onset and was not effective after hypoxaemia developed in randomized clinical trials [299]. Additional treatment trials are still ongoing or being planned to ascertain the clinical effectiveness of clinically approved drugs discovered in drug repurposing studies such as ivermectin, umifenovir, favipiravir, camostat, nafamostat, teicoplanin, and bismuth compounds [223, 300, 301].

While currently available antivirals have not demonstrated survival benefit, several immunomodulators have been shown to improve survival. Dexamethasone has been shown to reduce mortality by about 30% in patients requiring oxygen supplementation [302, 303]. Baricitinib, an inhibitor of Janus kinase, was shown to improve survival in patients treated with remdesivir, with a hazard ratio of death of 0.65 [304]. Conflicting or preliminarily positive results regarding the use of histamine receptor 2 antagonist famotidine, vitamin D, IL6 inhibitor tocilizumab and colchicine were reported [305–307]. Fluvoxamine, a selective serotonin reuptake inhibitor with high affinity for σ-1 receptor appeared to prevent clinical deterioration when given as early treatment for mild COVID-19 [308]. An open-labelled randomized trial showed that patients treated with recombinant human granulocyte colony stimulating factor have a lower risk of progressing to acute respiratory distress syndrome, sepsis, or septic shock [309]. Additional therapeutic approach that may include the manipulation of complement, neutrophil trapping function and TNF function are being discussed. More definitive large randomized control treatment trials are needed to confirm the usefulness of these immunomodulators.

Despite respiratory support by non-invasive ventilation by bilevel positive airway pressure or continuous positive airway pressure, some patients will still deteriorate and necessitate intubation and mechanical ventilation. In those who failed positive end expiratory pressure and prone ventilation, extracorporeal membrane oxygenation is the last step to support the patient till spontaneous recovery [310].

## Public health measures

We have shown that different epidemic waves in Hong Kong Special Administrative Region were due to different imported lineages of virus which became dominant during the epidemic surge and then disappeared with successful implementation of epidemiological control measures. Successful epidemic control depends on stopping case importation, minimizing community dissemination by social distancing measures, early detection and isolation of cases by extensive testing, rapid contact tracing and quarantine, and individual protection by universal masking and diligent hand hygiene. The resulting reduction of case load will protect our hospital and intensive care unit from paralysis and prevent the burnout of healthcare workers. Control at the border depends on minimizing the number of flights from highly epidemic areas with dangerous virus mutants, and testing all incoming travellers with no exemption, enclosed transportation and quarantining them for 14–21 days till negative surveillance testing. During the severe winter epidemic, city and even nation-wide lockdown with curfew to prevent gatherings is useful to enforce social distancing. The alternative way is to close or reduce the time of opening and occupancy of high-risk premises such as eateries, bars and fitness clubs where masks are often taken off. With sporadic clusters, district closure with mandatory RT–PCR testing of everyone followed by another testing at day 5–14 can be useful in stopping community transmission. Universal masking when outside home is demonstrated to stop the asymptomatic infected individual from shedding virus and to prevent susceptible individuals from acquiring infection as hinted by the hamster model [90]. Although surgical masks only have a fairly high effectiveness in blocking aerosols in the micron size range [311], it appears to be nearly as effective as N95 respirator [312]. Universal masking is shown useful in community epidemiological studies [313, 314]. Every case of unexplained fever or respiratory symptom should undergo mandatory testing. Repeated testing is indicated if the symptom persists as false negative may happen. While asymptomatic infection does occur, more than 80% of patients develop symptoms during the course of illness [315]. Moreover, only around 10% of infected persons are responsible for 80% of SARS-CoV-2 transmission. Thus catching this 10% by rapid multilayer contact tracing, early testing and quarantine of close contacts may identify the related asymptomatic or presymptomatic cases to stop further transmission. Rapid multilayer contact tracing, including non-close contacts and contacts of close contacts going back to more than 2 days before symptom onset, may be value added. Such labour-intensive contact tracing can be facilitated by a trained team with artificial intelligence analysing data of mobile phone applications or electronic payment. But these should be conducted in a manner to protect individual privacy [316]. Timely risk communication and education through media and internet are extremely important to secure cooperation from the public to make epidemic control a success.

## Infection control

The key measures of infection control against nosocomial outbreaks of COVID-19 include a combination of active surveillance for early case identification, isolation of suspected and confirmed case in the airborne infection isolation room (AIIR) with the implementation of standard, contact, droplets, and airborne precautions, as well as contact tracing to identify the potential secondary cases [317–320]. These infection control measures which had been proven to be effective in controlling SARS in 2003 were not as successful for COVID-19 [321], because asymptomatic infection contributes to a significant part of transmission and that the viral load peaks around the time of symptom onset. Thus universal screening of all hospital admissions or outpatient attendance by RT–PCR is warranted to reduce the risk of healthcare-related outbreaks. The risk for nosocomial transmission is especially high when asymptomatic COVID-19 patients are placed in non-AIIR rooms, or/and put on high-flow oxygen or non-invasive ventilation [322]. Therefore, universal masking for healthcare workers and hospitalized patients, if not medically contraindicated, in the clinical areas should be enforced to reduce the risk of COVID-19 transmission by respiratory droplets and short-range airborne route [323]. In fact, universal masking in the clinical areas can achieve zero nosocomial transmission of other respiratory viruses such as influenza A, influenza B, and respiratory syncytial virus [324]. Universal masking in the community also reduced the incidence of COVID-19 in the general population [325].

The overwhelming burden of hospitalized COVID-19 patients is another risk factor of nosocomial outbreaks. Alternative hospital sites such as temporary shelter hospital and convention halls have been built or re-purposed in mainland China, Hong Kong Special Administrative Region, the UK, the USA and Singapore as temporary measures to meet sudden surge in COVID-19 [326–328]. The infection control logistics and workflow in these alternative sites should be carefully planned and implemented to minimize the risk of outbreak [327]. The ventilation system of these alternative sites, especially the convention hall, was difficult to match with the hospital standard of 6–12 air changes per hour. Another parameter of ventilation by volume of air per second per person of around 60 L/s/person was considered acceptable as recommended by World Health Organization [329].

Appropriate use of personal protective equipment (PPE) is associated with a decreased risk of COVID-19 [330]. Full PPE includes use of N95 respirator, cap, face shield, gloves, and isolation gown of ASTM levels 1–3 were recommended [331]. However, critical shortage of PPE, especially N95 respirator, was a global problem during the initial phase of pandemic [332]. Reprocessing of N95 surgical respirator for reuse in performing aerosol generating procedures was also supported by the IDSA expert panel [333]. The methods of reprocessing include the use of vaporized, plasma, ionized hydrogen peroxide, ultraviolet radiation, and steam sterilization [333]. Quantitative fit test of N95 mask was performed to determine the maximum frequency of reprocessing [333].

Infection control training for proper donning and doffing of PPE is of utmost importance. Directly observed donning and doffing was promoted to maximize the protection and reduce the risk of self-contamination [334]. Simulation training has been used to enhance competency and alertness of healthcare workers, especially on the performance of high-risk procedures such as cardiopulmonary resuscitation [335].

## Animal models

SARS-CoV-2 probably evolved from an ancestral bat virus and jumped to humans via an unknown intermediate host [5]. SARS-CoV-2-related bat coronaviruses have now been found outside China, including Cambodia [336], Thailand [337], and Japan [338]. Over the course of the pandemic, it has become increasingly clear that SARS-CoV-2 has the potential to infect a wide range of animals. Natural human-to-animal transmission events involving dogs, cats, lions, tigers, and minks have been reported [71, 339–341]. Surrogate entry assays suggest that the S glycoprotein of SARS-CoV-2 has wide tropism for a variety of mammalian ACE2 receptors [342]. Therefore, it is not surprising that efficient animal models for COVID-19 could be rapidly established [343]. The first of these was the golden Syrian hamster (*Mesocricetus auratus*), which was quickly identified as a suitable model based on molecular docking analysis of its ACE2 with the SARS-CoV-2 RBD [89]. The clinical features of COVID-19 in human are well replicated in hamsters, which demonstrate a mild-to-moderate disease course with histopathological evidence of pneumonia. Therefore, hamsters are ideally suited to study the pathogenesis of SARS-CoV-2. Viral load dynamics in infected hamsters echo those of humans. Hamsters are able to transmit disease to each other via contact or non-contact transmission, thereby facilitating transmission studies [344]. The key limitation is the relative paucity of specific antibodies for detecting hamster biomarkers.

Other small animal models for SARS-CoV-2 research include ferrets (*Mustela putorius furo*) and mice (*Mus musculus*). Ferrets have a long pedigree of use in influenza research and are also susceptible to SARS-CoV-2 although the disease phenotype is quite mild and predominantly restricted to the upper respiratory tract [345, 346]. Given their convenience, mice models have also been developed, although this requires either virus adaptation to mouse ACE2 or humanized ACE2-expressing mice [343]. These have the disadvantage of modifying the disease phenotype, especially in human-ACE2 transgenic mice which have mild respiratory but severe brain disease. Depending on the promotor used, these human ACE2-transgenic mice exhibit variable phenotypes, ranging from mild disease to severe disease with encephalitis and even death [347, 348]. Mice humanized with human ACE2 using CRISPR/Cas9 knockin technology supports SARS-CoV-2 replication in the respiratory tract and brain tissues but generally develop only mild to moderate disease [349]. Adenovirus or adeno-associated virus-transduced mice develop self-limiting viral pneumonia, but has the advantages of being easy to generate and could be quickly adapted for different mouse strains [350, 351]. Laboratory rabbits can be infected with asymptomatic virus shedding [352].

As the ultimate origin of SARS-CoV-2 is likely to be from bats, one group has also demonstrated efficient infection of a fruit bat model (*Rousettus aegyptiacus*) with the virus. Fruit bats showed minimal clinical features of infection, but were capable of transmitting infection [353]. Notably, pigs and chickens, which are in close contact with humans, are not able to support productive infection, thus ruling them out as intermediate hosts [353, 354].

Small animal models such as those described above are convenient, but definitive evaluation of pathogenesis, antivirals and vaccines requires non-human primate models. Rhesus macaques (*Macaca mulatta*), cynomolgus macaques (*Macaca fascicularis*), African green monkeys (*Chlorocebus aethiops*), and baboons (*Papio*) are all susceptible to COVID-19 [355, 356]. Disease in non-human primates is typically mild, but disease severity and viral shedding increases with age as in humans.

## Vaccines

Over 70 SARS-CoV-2 vaccines developed from different vaccine technology platforms including inactivated whole virion, live attenuated virus, nucleic acid, virus vectors, and recombinant S protein, are already in clinical trials. Four vaccine candidates have published their phase 3 clinical data. While they all appear safe in clinical trials, each has its merits and demerits. The mRNA lipid nanoparticle vaccines induce good serum neutralizing antibody and cell-mediated immunity but requires stringent cold storage at −20 to −70°C [357]. Though this is a new technology, side effects are generally mild. Rare cases of anaphylaxis, possibly due to polyethylene glycol, have been reported after millions of doses have been administered [358]. Concerns of vaccine exacerbation of underlying medical illness in frail elderly aged over 80 years are not yet substantiated. The chimpanzee adenovirus and human adenovirus 26/5 vectored vaccines also induce high titres of neutralizing antibody and strong cell-mediated immunity with at least 70% vaccine efficacy [359, 360]. Further analysis of a phase 3 clinical trial showed that an adenovirus-vector-based vaccine was more effective if the interval between the first and second dose was 12 weeks or longer [361]. While the phase 3 clinical data from the beta-propiolactone inactivated whole virion vaccine have not yet been published in peer reviewed journals, the data from phase 2 trials suggested that the vaccine is safe and can induce neutralizing antibodies, but the data on cell-mediated immunity is limited at this stage [362, 363]. All three kinds of vaccines are likely to prevent severe symptomatic infection, but may not be able to prevent upper airway infection or transmissions, and are not well tested in children or pregnancy. The saponin-based recombinant trimeric spike nanoparticle appears to induce the best serum neutralizing antibody and reasonable cell-mediated immunity but phase 3 clinical trial data have not been published [364]. However, there are preliminary evidence that spike RBD virus mutants from South Africa and Brazil with E484 K mutation may reduce the neutralizing antibody titres induced by these vaccines [365, 366]. But as long as these vaccines protect vaccine recipients from severe disease, SARS-CoV-2 may just become another circulating common cold coronavirus when most of the global population has developed herd immunity by natural infections, or vaccination against the early Wuhan-related virus strains. Initial animal studies and phase 3 vaccination trials did not reveal any vaccine enhanced disease or antibody-dependent disease enhancement [367]. Instead, vaccination within 3 days before or after virus challenge in hamsters still showed varying degree of protection despite the lack of detectable neutralizing antibody titre at that juncture [367]. To maximize protection of the available vaccines, further studies on the effects of prime and boost approach by different combinations of vaccines are warranted. With the increasing availability of safe and effective vaccines, the battle is to fight misinformation and vaccine hesitancy by strategic education and risk communication so as to achieve a herd immunity of 70–80%.

## Epilogue

Emerging coronaviruses from animals have caused SARS in 2002–2003, MERS in 2012, and COVID-19 in 2019. These viruses have probably originated in bats and gone through intermediate wild mammals before jumping into humans. We predicted in 2007 that “the presence of a large reservoir of SARS-CoV-like viruses in horseshoe bats, together with the culture of eating exotic mammals in southern China, is a time bomb. The possibility of the reemergence of SARS and other novel viruses from animals or laboratories and therefore the need for preparedness should not be ignored” [368]. Spillover of SARS-CoV-2 from animals to humans appears to have happened in 2019. But unlike the other two highly pathogenic coronaviruses, the highly transmissible SARS-CoV-2 is able to overwhelm the healthcare system, inflict psycho-physical morbidities and mortalities, and disrupt our socioeconomic activities. More extensive and sustained animal surveillance for novel coronaviruses, monitoring of their evolution, and assessment of their risk of species jumping should be performed to understand the origin of SARS-CoV-2, the intermediate animal host, and to prepare for the next epidemic. The functions of many NSPs and ORFs of SARS-CoV-2, and their roles in viral life cycle and pathogenesis, are still uncertain. Unlike SARS which is usually quite symptomatic, the viral and immunological mechanisms underlying the generally milder symptoms or lack of symptoms in COVID-19 warrant more investigations. The types of samples and tests which can provide rapid, inexpensive and accurate diagnosis still need more research and development. With the early peaking of viral load, any effective antiviral strategy must be able to suppress the viral load sharply and coupled with immunomodulatory agents in order to improve the clinical outcome. Close monitoring of viral variants with increased virulence, transmissibility, and resistance to antivirals, antibodies or vaccines is important to combat this pandemic. The duration of protection by natural infection or vaccination, and the relative contribution to protection by neutralizing antibody or cell mediated immunity are still uncertain. Understanding the relative importance of transmission by droplet, aerosol, contact, and oral ingestion would provide more evidence to support recommendations on infection control measures and biosecurity standards of markets. Reusable, self-disinfectable and personalized filter-mask, eye protection, gloves and gowns should be developed as essential components of an environmental-friendly epidemic combat kit for everyone in the global village. Much more work by a highly coordinated real-time global surveillance network has to be done to win this war against COVID-19 and further emerging epidemics.
